# Association of hepatitis B vaccine response to vitamin D supplementation and ultraviolet B (UVB) exposure during different time intervals in experimental animals

**DOI:** 10.1007/s12026-022-09287-8

**Published:** 2022-05-19

**Authors:** Sara Youssry, Thanaa Shalaby, Al-Shaimaa Maher, Hossam Ghoneim

**Affiliations:** 1 Department of Immunology and Allergy, Medical Research Institute, Alexandria University, 165 El-Horreya Avenue, El Hadara, Alexandria, 21561 Egypt; 2 Department of Medical Biophysics, Medical Research Institute, Alexandria University, 165 El-Horreya Avenue, El- Hadara, Alexandria, 21561 Egypt; 3 Faculty of Science, Alexandria University, Alexandria, Egypt

**Keywords:** HBV, Vitamin D, UVB, Immunoglobulins, Avidity

## Abstract

The implications of vitamin D deficiency on the immune system have become clearer in recent years, being associated with less immune response following HBV vaccine. We aimed to elucidate the effect of vitamin D supplementation and UVB exposure on short- and long-term performance of hepatitis B vaccine. Forty-five male rabbits were randomly divided into 3 groups that were immunized with recombinant HBsAg. The first group (group I) represented a negative control group, whereas group III rabbits were administered with commercially available 1,25 (OH)2 vitamin D as an alternative for UVB exposure in group II. Results showed that vitamin D concentrations were significantly higher in UVB exposed group compared to both negative control and vitamin D-supplemented groups during short- and long-time intervals. In addition, means of anti-HBsAg isotypes’ levels and anti-HBsAg IgG avidity% were significantly higher in negative control group compared to other groups during short- and long-time intervals. Moreover, vitamin D serum concentration was positively correlated with anti-HBsAg IgG level and avidity % in both negative control and vitamin D-supplemented groups, while it was negatively correlated with anti-HBsAg IgM level in negative control group. It can be concluded from the above results that UVB radiation may have both augmenting and suppressive effects and that circulating serum vitamin D concentration may have a positive association with premium immune modulation following HBV vaccination.

## Introduction

Hepatitis B virus (HBV) infection is a major global health problem with approximately 300 million chronic HBV-infected individuals worldwide [1]. It is associated with up to 900,000 deaths every year mostly due to cirrhosis and HCC [2]. In Egypt, the frequency of positive HBsAg among general populations is about 1.5% [3]. Although the implementation of effective vaccination programs has resulted in a marked decrease in the incidence of new hepatitis B infection, it still remains a crucial cause of morbidity and mortality representing a major threat [4].

The development and maintenance of an efficient HBV-specific adaptive immune response represent the most important factors that can discriminate between HBV control and chronicity where B cells have been increasingly recognized to play an important role in the continuous control of HBV infection [5].

Hepatitis B vaccination has formerly been shown to be shaped by genetics and lifestyle factors [6], where there is widespread inter-individual variability in the magnitude of the antibody response after the second vaccination [7]. In addition, it has been observed that 10–15% of adults showed insufficient response by producing too few antibodies, as dictated by an anti-hepatitis B surface antigen immunoglobulin G (IgG) concentration of less than 10 mIU/mL [8].

The importance of vitamin D in the regulation of immune responses has been revealed such that achieving vitamin D sufficiency may be an important factor for the development of proper vaccine responses and consequently public health [9]. A significant moderate correlation was observed between vitamin D status and immune response to BCG vaccine [10], whereas other study revealed that serum level of vitamin D does not affect the immunogenicity of influenza vaccination in the elderly [11]. It has been reported that vitamin D levels were inversely correlated with HBV-DNA loads being associated with the clinical courses of HBV infection [12]. However, the impact of vitamin D on the development of the hepatitis B vaccination antibody response needs further exploration [13].

The current study aimed to elucidate the effect of oral vitamin D supplementation and ultraviolet B (UVB) exposure on the performance of hepatitis B vaccine throughout different time intervals by assessment of anti-hepatitis B surface antigen immunoglobulins’ (IgM, IgG and IgA) levels and IgG avidity % in experimental animals.

## Materials and methods

### Experimental animals

The present study was conducted on a total of 45 rabbits (1.2–1.3 kg body weight and 9–10 weeks) that were obtained from the farm of Faculty of Agriculture, Alexandria University, Egypt. The rabbits were acclimatized for 1 week prior to the experiment in the animal house (one rabbit per each battery) of Medical Research Institute, Alexandria University. Animals had free access to chow diet (standard rabbit food pellets) and water at room temperature.

### Ethical statement

All the experiments fulfil the guidelines of the National Institutes of Health guide for the care and use of Laboratory animals (NIH Publications No. 8023, revised 1978) and recommendations of Egypt’s guide for the care and use of laboratory animals [14]. The current study follows the ARRIVE Guidelines for reporting animal research and a completed ARRIVE checklist is included.

### Experimental design

**Fig. 1:**
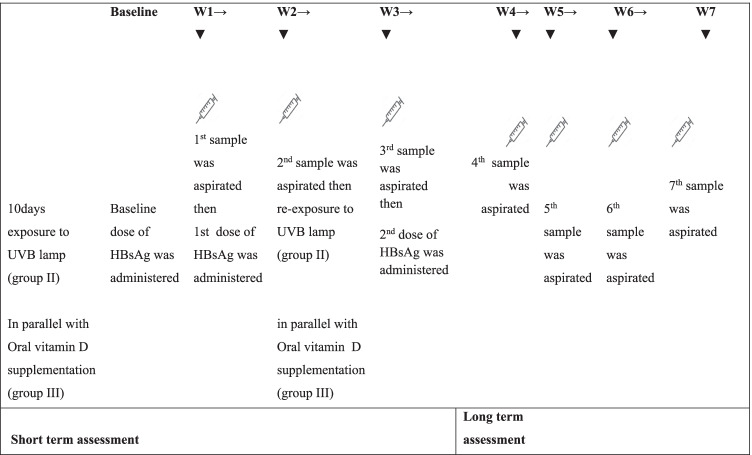
Experimental design

Rabbits were randomly divided into three groups (15 rabbits each) that were immunized with recombinant HBsAg (Euvax BLG Chem. 10 μg/0.5 ml; purchased from the Vacsera, vaccination centers in Cairo) at a dose (0.34 ug/kg of body weight) by intramuscular injection as a total of 3 doses (baseline, 1st and 2nd doses) at 0, 1 and 3 weeks sequentially. The first group represented a negative control group (without vitamin D support); the second group (UVB exposed) was exposed to UVB lamp for 10 days according to protocol of UV irradiation (15 min at a wavelength 254 nm, power 20 w and 30-cm distance) [15]; and the third group (vitamin D supplemented) was administered with vitamin D orally at a dose of 1680 IU/1.3 kg (calculated as 0.60 ml of the provided vial/250 ml drinking water) [16]. The 2nd and 3rd groups were exposed to UVB irradiation and administered with oral vitamin D, respectively, for 10 days before being immunized with vaccine dose, as shown in Fig. 1.

### Blood sampling

Fresh peripheral venous blood samples (2 ml) were obtained from ear veins in uncoated plain tubes. Samples were collected at baseline (i.e. week 0 at initial vaccination for vitamin D assessment) and at 1 week interval for 7 weeks following baseline HBsAg immunization dose (as a short- and long-term assessment). The blood was allowed to clot for 30 min at 37 °C followed by 1 h at 4 °C. After centrifugation, serum was then separated and collected in dry clean eppendorf tubes to be stored at − 80 °C until use for determination of total vitamin D serum concentrations as well as HBsAg antibodies’ levels and avidity.

### Serological assessments

#### Assessment of total vitamin D concentration by ELISA

Total vitamin D concentration was determined in sera from all the rabbits at baseline and at 1 week interval for 7 weeks after baseline HBsAg immunization dose using commercially available ELISA kit, according to the manufacturer’s instructions (abcam; Cat. No. ab213966).

#### Assessment of vaccine performance

##### Preliminary study for assessment of anti-HBsAg isotypes’ levels

HBsAg vaccine efficacy was monitored in sera collected from all the animals under study. Sera were assayed after each bleeding for anti-HBsAg specific classes of IgG, IgM and IgA by homemade ELISA using relevant HRP conjugated anti-rabbit IgG, IgM and IgA where the suitable concentrations for serum, coating HBsAg vaccine and HRP-conjugate were first identified [17]. Plates were initially coated with different concentrations of HBsAg (target vaccine) (0.5, 1, 2) µg/ml, serum samples (serially diluted from 1:10 to 1:200), and different dilutions of the conjugate (1:10,000 to 1:30,000). The absorbance of all wells was determined by an automated ELISA reader at a wavelength of 450 nm. The optical density (OD) was used to monitor the original level of anti-HBsAg specific classes in the test samples. Optimized working condition was as follows: 0.5 μg per well for vaccine coating, 1:100 of sample dilution and 1:10.000 of conjugate dilution.

##### Assessment of anti-HBsAg IgG avidity %

The strength of antigen–antibody interaction was assessed at various durations by incubation with denaturing agent (6-M urea) where pooled sera were prediluted 1:100 with a blocking buffer and dispensed in duplicates into wells coated with 0.5 μg/ml HBsAg [18]. The well contents were aspirated after incubation and were further incubated either in the presence or absence of 6-M urea where HRP-conjugated goat anti-rabbit IgG was then added to each well. The absorbance was determined by using EIA reader with wavelength set at 450 nm.

### Statistical analysis

Values were expressed as mean ± SD and were analysed using SPSS statistical software version 18 (SPSS, Chicago, IL). Multiple comparisons were performed using one-way ANOVA, followed by Tukey post hoc test. The correlation coefficients (*r*) between different assayed parameters were evaluated using Pearson correlation coefficient; *p* ≤ 0.05 was considered as the significance limit for all comparisons.

## Results

### Changes in vitamin D serum concentration in different groups during short- and long-time intervals of the study

The variations of vitamin D serum concentration along various time intervals in different groups are shown in Table 1. The mean of vitamin D serum concentration showed significant difference between different groups, where it was significantly upregulated in UVB-exposed group compared to both negative control and vitamin D-supplemented groups during both short- and long-time intervals of the study (*p* < 0.001). In addition, there was a significant difference in the kinetics of serum vitamin D concentrations throughout the experimental time in each group, where a relative increase was detected in negative control and vitamin D-supplemented groups throughout the experimental time. Regarding vitamin D concentrations in UVB-exposed group, a peak was observed in the 1st week followed by a gradual decline until the 5th week; however, another peak was detected in the 6th week.

**Table 1:** Comparison between the different studied groups according to serum vitamin D concentration

Vitamin D (ng/ml)	Negative control	UVB exposed	Vitamin D supplemented	*P*
Week 0	13.48 ± 1.01	29.69 ± 0.99	12.78 ± 0.92	< 0.001*
	*p*1 < 0.001*, *p*2 = 0.262, *p*3 < 0.001*			
Week 1	15.72 ± 0.79	35.11 ± 1.9	15.12 ± 0.85	< 0.001*
	*p*1 < 0.001*, *p*2 = 0.555, *p*3 < 0.001*			
Week 2	15.64 ± 1.37	29.64 ± 2.18	17.29 ± 0.73	< 0.001*
	*p*1 < 0.001*, *p*2 = 0.06, *p*3 < 0.001*			
Week 3	17.51 ± 0.64	29.25 ± 1.83	18.34 ± 0.65	< 0.001*
	*p*1 < 0.001*, *p*2 = 0.278, *p*3 < 0.001*			
Week 4	18.5 ± 0.67	25.08 ± 1.26	19.14 ± 0.79	< 0.001*
	*p*1 < 0.001*, *p*2 = 0.298, *p*3 < 0.001*			
Week 5	18.28 ± 0.56	28.42 ± 1.15	20.1 ± 1.07	< 0.001*
	*p*1 < 0.001*, *p*2 < 0.001*, *p*3 < 0.001*			
Week 6	21.07 ± 1.11	33.72 ± 1.98	21.75 ± 1.75	< 0.001*
	*p*1 < 0.001*, *p*2 = 0.634, *p*3 < 0.001*			
Week 7	20.7 ± 0.79	32.97 ± 1.61	19.91 ± 0.84	< 0.001*
	*p*1 < 0.001*, *p*2 = 0.287, *p*3 < 0.001*			
*p*0	< 0.001*	< 0.001*	< 0.001*	

*p: p *value for comparing between the different studied groups

*p*0: *p* value for comparing between different weeks in each group

*p*1: *p* value for comparing between Negative cont. and UV exposed

*p*2: *p* value for comparing between Negative cont. and vitamin D-supplemented group

*p*3: *p* value for comparing between UV exposed and vitamin D-supplemented group

^*^Statistically significant at *p* ≤ 0.05

### Changes in anti-HBsAg isotypes’ (IgG, IgM and IgA) levels in different groups during short- and long-time intervals of the study

**Fig. 2:**
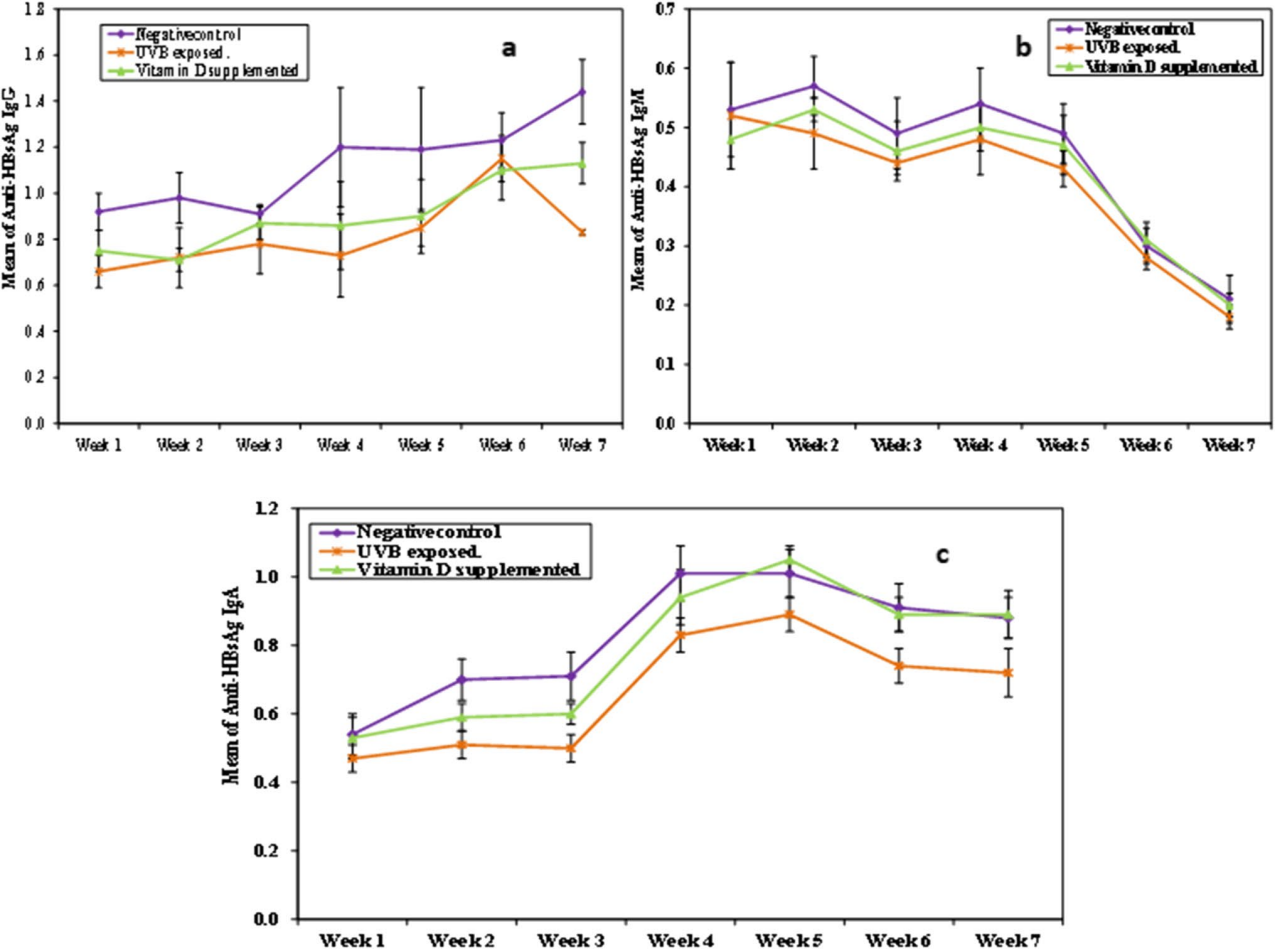
Kinetics of anti-HBsAg isotypes’ levels throughout experimental time in different groups after HBsAg immunization. (**a**) Kinetics of anti-HBsAg IgG levels, (**b**) kinetics of anti-HBsAg IgM levels, (**c**) kinetics of anti-HBsAg IgA levels

The changes of anti-HBsAg isotypes’ (IgG, IgM and IgA) levels along various time intervals in different studied groups are summarized in Table 2 and Fig. 2, where the results revealed that anti-HBsAg isotypes (IgG, IgM and IgA) levels were significantly higher in the negative control group compared to other groups during both short- and long-time intervals. There was also a significant difference in the kinetics of each isotype throughout the experimental time in each group, where serum anti-HBsAg IgM levels peaked in the 2nd and 4th weeks and then gradually declined throughout the experimental time, whereas anti-HBsAg IgG levels showed a relative increase in the studied groups throughout the experimental time; however, a decline was observed, following an increase, in UVB-exposed group in the 7th week. Regarding anti-HBsAg IgA levels, there was a constant increase till the 5th week followed by a gradual decline throughout the experimental time.

**Table 2:** Comparison between the different studied groups according to different anti-HBsAg isotypes’ (IgG, IgM and IgA) levels

	Anti-HBsAg IgG			*p*	Anti-HBsAg IgM			*p*	Anti-HBsAg IgA			*p*
	Negativecontrol	UVB exposed	Vitamin D supplemented		Negative control	UVB exposed	Vitamin D supplemented		Negative control	UVB exposed	Vitamin D supplemented	
Week 1	0.92 ± 0.08	0.66 ± 0.07	0.75 ± 0.09	< 0.001*	0.53 ± 0.08	0.52 ± 0.09	0.48 ± 0.05	0.355	0.54 ± 0.06	0.47 ± 0.04	0.53 ± 0.06	0.008*
	*p*1 < 0.001*, *p*2 < 0.001*, *p*3 = 0.089								*p*1 = 0.014*, *p*2 = 0.974, *p*3 = 0.024*			
Week 2	0.98 ± 0.11	0.72 ± 0.13	0.71 ± 0.05	< 0.001*	0.57 ± 0.05	0.49 ± 0.06	0.53 ± 0.02	0.003*	0.7 ± 0.06	0.51 ± 0.04	0.59 ± 0.04	< 0.001*
	*p*1 < 0.001*, *p*2 < 0.001*, *p*3 = 0.974				*p*1 = 0.002*, *p*2 = 0.119, *p*3 = 0.223				*p*1 < 0.001*, *p*2 < 0.001*, *p*3 = 0.003*			
Week 3	0.91 ± 0.04	0.78 ± 0.13	0.87 ± 0.07	0.009*	0.49 ± 0.06	0.44 ± 0.02	0.46 ± 0.05	0.099	0.71 ± 0.07	0.5 ± 0.04	0.6 ± 0.03	< 0.001*
	*p*1 = 0.008*, *p*2 = 0.560, *p*3 = 0.085								*p*1 < 0.001*, *p*2 < 0.001*, *p*3 < 0.001*			
Week 4	1.2 ± 0.26	0.73 ± 0.18	0.86 ± 0.19	< 0.001*	0.54 ± 0.06	0.48 ± 0.06	0.5 ± 0.04	0.068	1.01 ± 0.08	0.83 ± 0.05	0.94 ± 0.08	< 0.001*
	*p*1 < 0.001*, *p*2 = 0.005*, *p*3 = 0.374								*p*1 < 0.001*, *p*2 = 0.118, *p*3 = 0.009*			
Week 5	1.19 ± 0.27	0.85 ± 0.08	0.90 ± 0.16	< 0.001*	0.49 ± 0.05	0.43 ± 0.03	0.47 ± 0.05	0.023*	1.01 ± 0.07	0.89 ± 0.05	1.05 ± 0.04	< 0.001*
	*p*1 = 0.001*, *p*2 = 0.006*, *p*3 = 0.788				*p*1 = 0.018*, *p*2 = 0.398, *p*3 = 0.254				*p*1 < 0.001*, *p*2 = 0.257, *p*3 < 0.001*			
Week 6	1.23 ± 0.12	1.15 ± 0.10	1.10 ± 0.13	0.069	0.3 ± 0.03	0.28 ± 0.02	0.31 ± 0.03	0.076	0.91 ± 0.07	0.74 ± 0.05	0.89 ± 0.05	< 0.001*
									*p*1 < 0.001*, *p*2 = 0.630, *p*3 < 0.001*			
Week 7	1.44 ± 0.14	0.83 ± 0.01	1.13 ± 0.09	< 0.001*	0.21 ± 0.04	0.18 ± 0.02	0.2 ± 0.02	0.067	0.88 ± 0.06	0.72 ± 0.07	0.89 ± 0.07	< 0.001*
	*p*1 < 0.001*, *p*2 < 0.001*, *p*3 < 0.001*								*p*1 < 0.001*, *p*2 = 0.983, *p*3 < 0.001*			
*p*0	< 0.001*	< 0.001*	< 0.001*		< 0.001*	< 0.001*	< 0.001*		< 0.001*	< 0.001*	< 0.001*	

*p*: p value for comparing between the different studied groups

*p*0: *p* value for comparing between different weeks in each group

*p*1: *p* value for comparing between Negative cont. and UV exposed

*p*2: *p* value for comparing between Negative cont. and vitamin D suppl

*p*3: *p* value for comparing between UV exposed and vitamin D suppl

^*^Statistically significant at *p* ≤ 0.05

### Changes in anti-HBsAg avidity % in different groups during short- and long-time intervals of the study

**Fig. 3:**
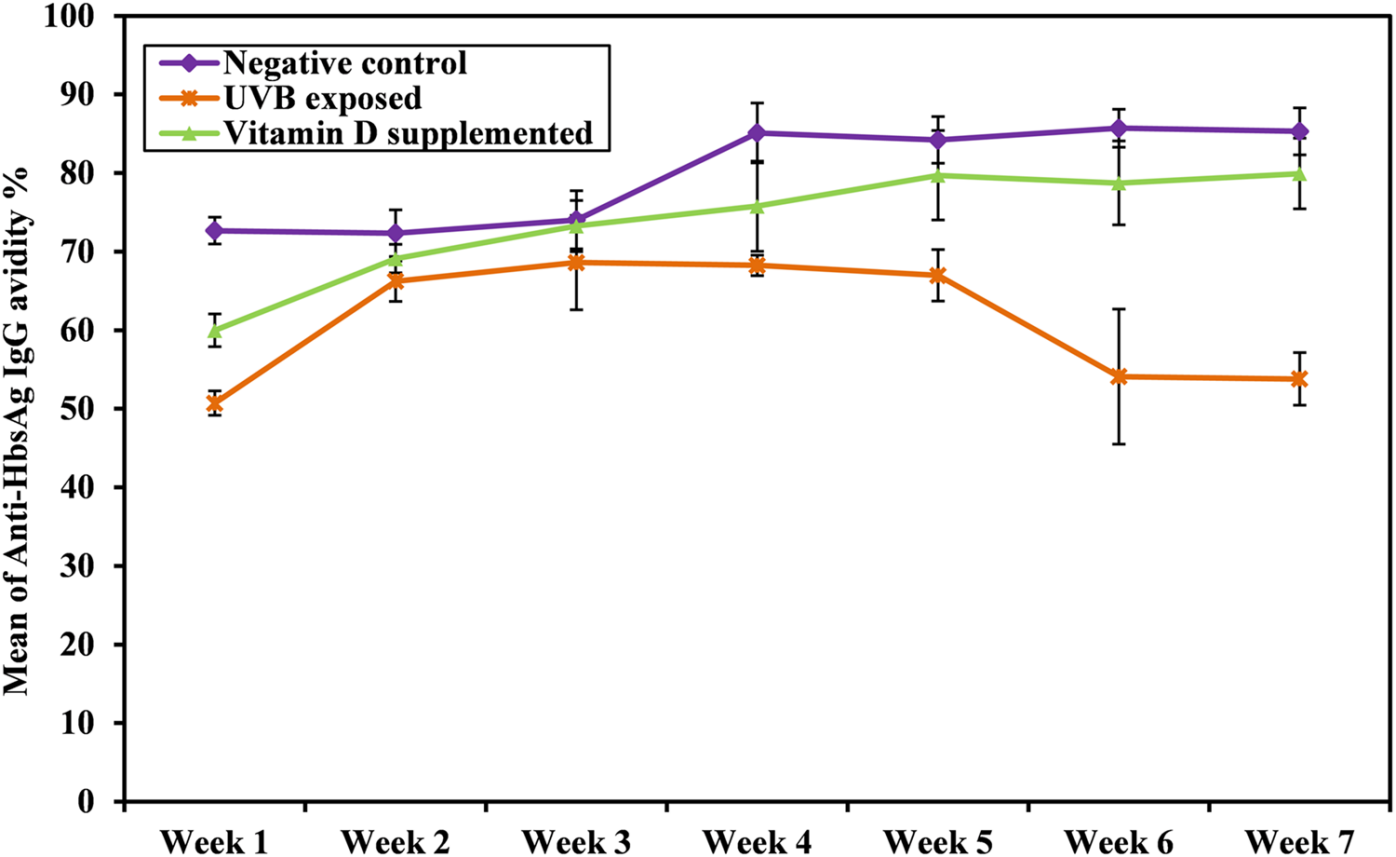
Kinetics of anti-HBsAg IgG avidity % in different groups throughout experimental time after HBsAg immunization

We further evaluated the strength of binding between the immunizing HBsAg and its corresponding anti-HBsAg IgG as an indicator of protective immunity after immunization with HBV vaccine. As shown in Table 3 and Fig. 3, the results reveal that mean anti-HBsAg IgG avidity% is significantly higher in the negative control group compared to the other groups during short- and long-time intervals. A significant difference was also observed in the kinetics of anti-HBsAg IgG avidity% throughout the experimental time in each group, where anti-HBsAg IgG avidity % showed a relative increase in negative control and vitamin D-supplemented groups throughout the experimental time; however, a gradual decline was detected following this relative increase in UVB-exposed group in the 5th week.

**Table 3:** Comparison between the different studied groups according to anti-HBsAg IgG avidity %

	Anti-HbsAg IgG avidity %			*p*
	Negative control	UVB exposed	Vitamin D supplemented	
Week 1	72.67 ± 1.69	50.71 ± 1.55	59.98 ± 2.1	< 0.001*
	*p*1 < 0.001*, *p*2 < 0.001*, *p*3 < 0.001*			
Week 2	72.35 ± 2.96	66.24 ± 2.57	69.1 ± 1.83	< 0.001*
	*p*1 < 0.001*, *p*2 = 0.018*, *p*3 = 0.045*			
Week 3	74.03 ± 3.7	68.61 ± 6	73.25 ± 3.25	0.025*
	*p*1 = 0.031*, *p*2 = 0.920, *p*3 = 0.071			
Week 4	85.1 ± 3.81	68.25 ± 1.3	75.78 ± 5.74	< 0.001*
	*p*1 < 0.001*, *p*2 < 0.001*, *p*3 < 0.001*			
Week 5	84.2 ± 2.97	66.97 ± 3.27	79.71 ± 5.7	< 0.001*
	*p*1 < 0.001*, *p*2 = 0.058, *p*3 < 0.001*			
Week 6	85.7 ± 2.4	54.1 ± 8.6	78.74 ± 5.33	< 0.001*
	*p*1 < 0.001*, *p*2 = 0.039*, *p*3 < 0.001*			
Week 7	85.3 ± 2.98	53.8 ± 3.36	79.93 ± 4.5	< 0.001*
	*p*1 < 0.001*, *p*2 = 0.008*, *p*3 < 0.001*			
*p*0	< 0.001*	< 0.001*	< 0.001*	

*p*: p value for comparing between the different studied groups

*p*0: *p* value for comparing between different weeks in each group

*p*1: *p* value for comparing between Negative cont. and UV exposed

*p*2: *p* value for comparing between Negative cont. and vitamin D suppl

*p*3: *p* value for comparing between UV exposed and vitamin D suppl

^*^Statistically significant at *p* ≤ 0.05

### Association between serum vitamin D concentration and anti-HBsAg isotypes in different groups during short and long time intervals of the study

To elucidate the effect of vitamin D supplementation and UVB exposure on short- and long-term performance of hepatitis B vaccine, we analysed the association between vitamin D serum concentration with both anti-HBsAg immunoglobulins’ levels and avidity% (Table 4). The results showed that serum vitamin D concentration was positively correlated with anti-HBsAg IgG in both negative control (*r* = 0.756, *p* = 0.049) and vitamin D supplemented (*r* = 0.765, *p* = 0.045) groups, whereas it was negatively correlated with anti-HBsAg IgM in negative control group (*r* =  − 0.775, *p* = 0.041). Regarding association with anti-HBsAg avidity %, vitamin D concentration was positively correlated with IgG avidity % in both negative control (*r* = 0.869, *p* = 0.011) and vitamin D-supplemented (*r* = 0.911, *p* = 0.004) groups.

**Table 4:** Correlation between serum vitamin D concentration and anti-HBsAg isotypes’ levels as well as anti-HBsAg IgG avidity% in the different studied groups

		Anti-HBsAg-IgG			Anti-HBsAg IgM			Anti-HBsAg IgA			Anti-HBsAg IgG avidity %		
		Negativecontrol	UVBexposed	Vitamin D supplemented	Negative control	UVB exposed	Vitamin D supplemented	Negative control	UVB exposed	Vitamin D supplemented	Negative control	UVB exposed	Vitamin D supplemented
Vitamin D concentration	*r*	0.756	0.211	0.765	− 0.775	− 0.329	− 0.47	0.613	− 0.411	0.729	0.869	− 0.672	0.911
	*p*	0.049*	0.649	0.045*	0.041*	0.471	0.287	0.144	0.359	0.063	0.011*	0.098	0.004*

*r* Pearson coefficient

^*^Statistically significant at *p* ≤ 0.05

## Discussion

Although HBV vaccination is the most effective approach to prevent HBV infection, there are still 5–10% of the subjects who fail to produce protective anti-HBsAg titer after three times of vaccination irrespective of the source of the antigen [19]. The underlying mechanisms of the poor immune responses have not been elucidated [20]. It has been hypothesized that vitamin D levels could have an impact on immune responses to vaccines [21]. However, few data are available concerning the relationship between vitamin D level and vaccine responses against hepatitis B [22].

The present study revealed a statistical significant difference in the mean of vitamin D serum concentration between the different groups, where it was markedly higher in UVB-exposed groups compared to both negative control and vitamin D-supplemented groups during short- and long-time intervals. Our results are in agreement with Kalajian et al. [23] who postulated that UVB light lamp is more effective in producing vitamin D3 than sunlight. It has been claimed that regular UVB exposure increases serum vitamin D concentration to levels exceeding that obtained through oral cholecalciferol by at least 20 µg [24]. In addition, other factors as strategies employed by health care providers including (type, dose and duration of vitamin D supplementation) and environmental factors may explain the decreased vitamin D levels in vitamin D-supplemented group [25].

We assessed different anti-HBsAg isotypes (IgG, IgM and IgA) levels as well as anti-HBsAg IgG avidity% to evaluate the role of vitamin D as a potential HBV vaccine adjuvant. The present study revealed that the means of anti-HBsAg isotypes (IgG, IgM and IgA) as well as anti-HBsAg IgG avidity% were significantly higher in immunized rabbits that did not receive extrinsic vitamin D support (negative control group) relative to their partners exposed to either UVB or supplemented with oral vitamin D3 during both short- and long-time intervals.

The remarkable reduction in anti-HBsAg isotypes following immunization of vitamin D supplemented rabbits (group III) may be explained on that the status of vitamin D at the time of initial vaccination may influence the subsequent secondary hepatitis B vaccine response where low vitamin D status at initial vaccination was associated with poorer vaccine response [26]. In addition, it has been observed that vitamin D supplementation beginning 3 days after the initial hepatitis B vaccination did not influence the hepatitis B vaccine response [27]. Moreover, the observed reduction in both antibodies’ levels and avidity in UVB-exposed group may be attributed to the possible immunosuppressive effect following exposure to UVR [28], where the observed upregulation of vitamin D status in UVB-exposed group could be a compensatory mechanism to overcome this immunosuppression.

Furthermore, our study revealed that vitamin D serum concentration was positively correlated with anti-HBsAg IgG levels and avidity in negative control and vitamin D-supplemented groups during short- and long-time intervals while it was negatively correlated with anti-HBsAg IgM levels in negative control group. It has been observed that high vitamin D level successfully boosters immunization-induced specific-antibody titers, where vitamin D may modulate vaccine responses through its interaction with antigen presentation, dendritic cell migration as well as the subsequent activation of T and B cell antibody responses[29].

In concordance, it has been shown that adult mice vaccinated subcutaneously or intramuscularly with inactivated vaccine co-administered with 1,25-(OH)2D3 enhanced systemic immune responses where vitamin D was reported to improve HBV vaccine's humoral immune response when used as adjuvant [30]. On the contrary, other study demonstrated that vitamin D supplementation promotes a higher TGF-β plasma level in response to vaccination without improving antibody production, while other study observed no correlation between vitamin D levels and vaccine responses [22].

Despite the immune-modulatory role of vitamin D in response to viral vaccines, the negative association with anti-HBsAg IgM may be related to Ig class-switch recombination which occurred with the beginning of vaccine intake from IgM towards IgG and IgA [31].

Although the present findings are notable, the study should be expanded by assessing vitamin D and UVB dose–response curve. It can be concluded from the above results that UVB radiation may act as a double edged sword in response to HBV vaccine response, since it is a major source of vitamin D; however, being associated with a reduced vaccine response. In addition, vitamin D may have a possible immunomodulatory role in the development of premium HBV–vaccine response.
